# Jefferson Medical College

**Published:** 1843-07-08

**Authors:** H. T. Child


### CLINICAL LECTURES AND REPORTS.

### JEFFERSON MEDICAL COLLEGE.

### CLINIC OF PROFESSOR MITCHELL.

*Dispensary of Jefferson Medical College, July* 5, 1843.

(Reported by H. T. Child.)

### LECTURE I.

CASE I.—Margaret McG——, æt 30. Intermittent fever, tertian type of six weeks standing, attended with constipation. Professor M. remarked that was a common case, and presented no peculiarities, and the treatment would be very simple. There is much more noise made in the medical profession about the effect of *different* doses of quinine than is warranted by these effects; in ordinary cases a medium dose is altogether sufficient to arrest the paroxysms. My usual practice in these cases is to give quiniæ sulphas, gr. v., and pulv. opii, gr. i., an hour previous to the expected return of the paroxysm, directing the patient to go to bed and promote sudoresis by warm clothing. There is a great liability to a return of this disease; to prevent this I am in the practice of giving small doses, say grs. iij. daily, for twenty-one days, when, if there be no organic lesion or renewal of the exciting cause, the patient will be well.

CASE II.—James D., æt. 35. Has had a cough for three weeks, accompanied with pain in the head, chest and lower extremities.

This, gentlemen, is a case of influenza, the prevailing epidemic, which is now exciting so much interest in the minds of medical men. Unlike most other epidemics which originate to the N. E., this commenced its ravages in New York. Perhaps the aerial poison was introduced into that place from the sea. We have no account of the existence of the epidemic at any other place previous to its appearance in that city. It appears to have travelled in all directions—having reached Boston about the same time that it appeared in this city.

The *causes* of these epidemics are involved in the greatest mystery. That the poison is either something superadded to, or detracted from the atmosphere, which is imperceptible, except by its effects, is very evident; the prevailing opinion is that the former is the case, and Dr. Prout has suggested the idea that the poison is thrown out from the earth by the operation of volcanoes and earthquakes. This suggestion is worth examination, particularly at the commencement of the investigation of this very important subject; for, said Professor M., when we shall have discovered the nature of this aerial poison, we shall no doubt be able to find means of counteracting and destroying its influence,—and the man who shall make this discovery, will be a greater benefactor than even the immortal Jenner.

*Symptoms.*—Being in New York about the time this disease first made its appearance, I had an opportunity of witnessing some of the most severe cases; so violent was it, at first, that several persons died before medical aid could be applied to arrest the disease. It assumes a great variety of forms, the most common cases commenced with violent pain in the frontal region, so severe, in some instances, as to produce delirium, this was followed in a few hours by pain in the malar region, extending up to the eyelids, after this there was a copious secretion from the schneiderian membrane, in many cases bloody, and so profuse that it ran in a stream. One fellow in New York said he felt as if he had a Croton pipe passing through him. These stages lasted from twelve to twenty-four hours, and, unless arrested by proper treatment, were followed by violent congestion of the lungs, which in turn was followed by profuse secretion; after this there was but little danger, except in those cases (which you know are so frequent in this country,) where the tubercles were lying dormant; the shock is almost certain to rouse them and lay the foundation for permanent and serious pulmonary affections. In this case as in other epidemics, those persons who were attacked early in the season would have a cough more or less severe throughout the prevalence of the epidemic; and he remarked to a patient of his that when the last man who took the influenza got clear of his cough he might then expect to, but not before. In Boston and in this city it had not been so severe as in New York—among the anomalous symptoms might be ranked, muscular pains, most frequently of the neck; the lower extremities were often painful, and there was a general sense of weakness, and a profuse clammy perspiration, so that the beds and bedding were soaked. Unlike most other epidemic influenza which have attacked children and old persons, this has been more severe upon the middle aged and those of strong, robust constitution.

*Treatment.*—This of course varied according to different views of practitioners. Some who consider that epidemics will run a definite course, were disposed to use a placebo—flaxseed tea or mild syrup, and warm pediluvia. Others recommend the use of emetics, cathartics, &c. &c., but, gentlemen, the practice upon principle in this case, as in all others, is the only one that I would recommend. Treat the symptoms as they exist; and whether it be in epidemic or sporadic cases, your success will be greater than under any other plan. That the influence of an epidemic does operate against the successful treatment of any disease, we all know. How would you be likely to succeed in resuscitating a drowning man while he is still under the water? Go forward, then, upon principle, and you will be able boldly to combat this disease. In many cases I have found abstraction of blood, either by cups or leeches, or general bleeding, to afford speedy and permanent relief. Topical bleeding, I generally prefer; and strange to say, in the sweating stages no remedy has been found so successful as sudorifics. Instead of the cold and clammy perspiration in which the patient is bathed, you will find, under the use of acidulous drinks, neutral mixture, tartar emetic, &c., that the natural and healthy exhalations of the cutaneous surface will be restored. A mercurial cathartic followed by Saratoga water, salts, or any other purgative, may also be prescribed with advantage. Under this treatment persons who, from the severity of the first symptoms, supposed they were to be sick several weeks, have in a day or two been able to attend to their ordinary avocations.

We will have the patient before you cupped over the chest—a purge of Pilul Hydrarg., and to-morrow follow it by Magnes. Sulph. ℥j.

CASE III.—E. H. æt. 30. Pain in the chest, and cough. Treatment as before, with a blister over chest.

CASE IV.—Robt. P——, æt. 26. Influenza. Treatment—Venesection, ℥xii.; Mercurial purge, followed by salts.

CASE V.—John T——, æt. 60. Nervous debility; great weakness and tremors of the extremities; atrophy of the nervous centres.

This case, gentlemen, we shall treat homœopathically—not Hahnnemanically. Homœopathy is as old as Hippocrates—but we shall not give him a fraction of the shadow of the shade of the ghost of a dose. Give him three grains of the iodide of potassium, three times a day in a wine glassful of sweetened water. This remedy has been noted for producing atrophy of different organs.

Case vi.—Francis McL——, æt. 60. Chronic gastritis; dyspepsia; pulse full, active. Treatment—Venesection and Pills of Aloes Rhei, Calomel and Soap, one daily.

Case vii.—Chas. McG——, æt. 31. Influenza. Treatment as in the other cases.

Case viii.—Ann W——, æt. 40. do. do.

Case ix.—Mary Ann G——, æt. 42. Chronic pain around the anterior fontanel; periostitis. Treatment—Counter-irritation by Ung. Ant. et. Pot. Tart. immediately over the affected part.

Case x.—Archy R——, æt. 70. Hematemesis, with general prostration. This, said Prof. M., is a case of the wearing out of the system, and in which we can do but little. We will give him some diffusible stimulant—say two grains of sesqui-carbonate of ammonia, and five drops of aromatic spirit of ammonia, in solution, twice a day.

Case xi.—Mary E——, æt. 30. Has had intermittent fever; complains of worms, which she has seven times ejected from the stomach. Treatment—Spirits Terebinth. gtts. xx.; Oleum Ricini ℥ss. every morning.

Case xii.—Julia L——, æt. 45. Influenza. Treatment as before.

Note. There were seven or eight other cases prescribed for at this clinic.

